# Efficacy and safety of electroacupuncture in patients with postpartum depression: a meta-analysis

**DOI:** 10.3389/fpsyt.2024.1393531

**Published:** 2024-07-11

**Authors:** Xue Fang, Xiaoyan Wang, Wenjun Zheng, Jing Han, Xiaobin Ge

**Affiliations:** ^1^ Department of Acupuncture and Moxibustion, Affiliated Hospital of Shandong University of Traditional Chinese Medicine, Jinan, Shandong, China; ^2^ College of Acupuncture and Massage, Shandong University of Traditional Chinese Medicine, Jinan, Shandong, China; ^3^ School of Basic Medical Sciences, Shandong First Medical University & Shandong Academy of Medical Sciences, Jinan, Shandong, China; ^4^ Department of Acupuncture-Moxibustion and Tuina, Qilu Hospital of Shandong University, Jinan, Shandong, China

**Keywords:** postpartum depression, electroacupuncture, HAMD, EPDS, meta-analysis

## Abstract

**Objective:**

This study aimed to assess the clinical effectiveness and safety of electroacupuncture (EA) for the treatment of postpartum depression (PPD).

**Methods:**

We systematically retrieved relevant randomized controlled trials (RCTs) from electronic databases, including PubMed, Cochrane Library, China National Knowledge Infrastructure, EMBASE, China Science and Technology Journal Database, Chinese Biological Medical Database, and the Wanfang database from their inception to November 2023. The outcomes measured were the Hamilton Depression Rating Scale (HAMD) scores, Edinburgh Postnatal Depression Scale (EPDS) scores, adverse events (AEs), and the total response rate. The study aimed to estimate heterogeneity, publication bias, mean difference (MD), and risk ratios (RR) with 95% confidence intervals (CIs).

**Results:**

This study included 12 RCTs with a total of 1364 participants (571 in the treatment group and 793 in the control group) for analysis. The results of the meta-analysis indicated that EA did not significantly reduce HAMD (MD = 1.49, 95% CI = [−0.30, 3.27], P = 0.1, *I^2^* = 0%) and EPDS (MD = 1.12, 95% CI = [−1.62, 3.85], P = 0.42, *I^2^* = 32%) scores compared to sham EA among patients with PPD, resulting in low heterogeneity. In terms of the total response rate, the EA group exhibited superior results compared to the placebo group (RR = 1.77, 95% CI = [1.15, 2.74], P = 0.01, *I^2^* = 0%) and the sham EA group (RR = 1.2, 95% CI = [1.02, 4.4], P = 0.02, *I^2^* = 0%), with statistical significance and low heterogeneity. The incidence of AEs was lower, also with low heterogeneity (RR = 0.9, 95% CI = [0.57, 1.43], P = 0.66, *I^2^* = 12%).

**Conclusion:**

The current evidence indicates that the effectiveness and safety of EA in the treatment of PPD warrant affirmation. However, EA does not demonstrate superiority over sham EA in reducing HAMD and EPDS scores in patients with PPD. Due to the limited quantity and quality of curent research, the above conclusion should be further validated through high-quality studies to confirm the efectiveness of EA in PPD management.

**Systematic review registration:**

https://www.crd.york.ac.uk/PROSPERO/display_record.php?RecordID=318917, identifier CRD42023318917.

## 1 Introduction

Postpartum depression (PPD) is a significant mental health issue that has detrimental effects on both mothers and their infants. It is characterized by evident depressive symptoms during the postpartum period, mainly manifesting as feelings of depression, lethargy, and an increased tendency to cry (1). According to a recent meta-analysis encompassing 565 studies from 80 different countries or regions, the global incidence rate of PPD was estimated to be approximately 17.22% (2). Between 10% and 20% of women are at risk of experiencing PPD within the first year after childbirth (3). In addition to the physical discomfort and functional limitations experienced by women after giving birth, PPD exerts a negative impact on the offspring of the affected mothers. Studies have shown that PPD can restrict a woman’s capacity to effectively perform her maternal duties, leading to suboptimal care for her infant (4–6).

Currently, the primary approach for treating PPD involves drug therapy, specifically the use of medications such as fluoxetine and sertraline (7, 8). Despite the minimal effects of these drugs on breastfeeding, concerns persist among breastfeeding mothers. Studies have associated the use of these medications with irritability and sleep disturbances, particularly in premature or low-birth-weight infants (9, 10). In addition, abruptly discontinuing these medications is detrimental to the well-being of the mother and the newborn, leading to numerous adverse effects. An alternative approach to the treatment of PPD involves nonpharmacological methods. Cognitive behavioral therapy (CBT) has been recommended as an evidence-based psychotherapy by clinical practice guidelines for the majority of individuals with PPD (11). Although CBT offers a promising approach, it is essential to take into account the challenges associated with compliance with it (12).

Electroacupuncture (EA) therapy is a form of acupuncture used in complementary and alternative medicine. Its fundamental principle involves the application of electrical current at specific acupoints to regulate the physiological function of the human body, with the aim of treating diseases. Compared to manual acupuncture, EA offers improved control over stimulation parameters, as well as greater repeatability and objectivity (13). There is emerging evidence indicating the therapeutic potential of EA in addressing psychological disorders such as anxiety and depression (14–17). EA has also been used in PPD treatment, showing specific therapeutic benefits. Nevertheless, there is currently a dearth of meta-analyses and systematic reviews on the effectiveness of EA for PPD. The objective of this study is to evaluate the evidence of EA for PPD through a meta-analysis of randomized controlled trials (RCTs).

## 2 Materials and methods

The Cochrane Handbook for Systematic Reviews of Interventions was used to conduct this meta-analysis, and the Preferred Reporting Items for Systematic Reviews and meta-analyses guidelines were used to describe the results (**Supplementary Table S1**). The meta-analysis protocol (CRD42023318917) was found in the PROSPERO database.

### 2.1 Search strategy

To retrieve pertinent articles published up to November 2023, two independent researchers conducted searches on electronic databases, including PubMed, Cochrane Library, China Science and Technology Journal Database (VIP), China National Knowledge Infrastructure (CNKI), Chinese Biological Medical Database (CBM, SinoMed), Wanfang database, and EMBASE. The search included the terms “electroacupuncture” and “Postpartum Depression”. **Supplementary Table S2** presents a comprehensive list of the search techniques utilized in this study.

### 2.2 Inclusion criteria

The studies had to meet specific criteria in order to be eligible for inclusion in this review. The population consisted of individuals diagnosed with PPD, based on the criteria outlined in the Diagnostic and Statistical Manual of Mental Disorders (DSM) (18), the Chinese Classification and Diagnostic Criteria of Mental Disorders (CCMD) (19), as well as the Hamilton Depression Scale (HAMD), Edinburgh Postpartum Depression Scale (EPDS), and clinical manifestations. Interventions in the study groups included EA alone, EA combined with traditional Chinese medicine (TCM), and EA combined with antidepressants. There were no restrictions on the selection of acupuncture points, specific manipulation techniques, treatment duration, or frequency.

The control group employed treatment methods excluding EA intervention, such as sham EA; blank control; TCM; psychological intervention; and antidepressants such as paroxetine, sertraline, mirtazapine, and venlafaxine. Primary outcome indicators comprised the HAMD scores, EPDS scores, and the total response rate, whereas secondary outcomes involved adverse events (AEs). The study design was based on RCTs that provide comprehensive baseline data and comparative efficacy assessments, encompassing reports containing comprehensive information.

### 2.3 Data extraction

Two independent researchers (X.F. and X.B.G.) individually examined and calculated all the data. In cases of disagreement, the two investigators engaged in discussions to resolve their differences. If a consensus could not be reached, they consulted another investigator (X.Y.W.), and decisions were made based on a majority vote. All the involved researchers demonstrated proficiency in the administration of EA for the treatment of PPD.

The data presented in this study were obtained from individual clinical trials, including details such as the number of participants in the EA and control groups, demographic details (age), geographical location, study methodology, duration, interventions (such as type of acupuncture, duration, treatment frequency, and acupoint selection), and outcomes (**Table 1**). If the chosen reports lacked complete data, the authors were contacted via email. Studies that did not yield a response were excluded from our analysis. The third investigator was tasked with resolving any inconsistencies in the data.

**Table 1:** Basic characteristics of patients in the included studies.

Study	Country	Diagnostic criteria	Design	Experimental group							Control group				Outcome	Measurement timepoint (month)
				Sample size	Age (year)	Course of disease	Intervention	Acupuncture points	Duration of acupuncture	Acupuncture frequency	Sample size	Age (year)	Course of disease	Intervention		
Gong LP, (20)	China	EPDS	2, RCT	21	28.25 ± 3.42	NM	EA + paroxetine	baihui(DU20) yintang(EX-HN3)	20min	7 times a week for 8 weeks	21	27.21 ± 2.35	NM	paroxetine	①	2
Lin JX, (21)	China	Diagnostic and Statistical Manual of Mental Disorders (DSM) + clinical manifestations	2, RCT	34	30.06 ± 3.34	NM	EA + Guipitang	yintang(EX-HN3) baihui (DU20) Genital area (a 2-centimeter straight line parallel to the anterior and posterior midline upward from the frontal horn)	30min	5 times a week for 4 weeks	34	30.45 ± 3.41	NM	psychotherapy	①②③④	1
Liu XB, (22)	China	EPDS + clinical manifestations	2, RCT	165	NM	NM	EA + Xiaoyaosan	qimen(LA14) taichong(BL15) fenglong(ST40) sanyinjiao(SP6) xuehai(SP10) pishu(BL20) zusanli(ST36) tiantu(RN22) neiguan(PC6)	30min	7 times a week for 6 weeks	171	NM	NM	Sertraline Hydrochloride Tablets	⑤	1.5
Su W, (23)	China	Chinese Classification and Diagnostic Criteria of Mental Disorders (CCMD)	2, RCT	58	28.18 ± 3.24	NM	EA	yintang(EX-HN3) baihui (DU20) zhongwan(RN12) qihai(RN6)	30min	3 times a week for 8 weeks	58	28.02 ± 3.11	NM	Xiaoyaowan	①②③④⑥ ⑦⑧⑨⑩	2,3
Wang HZ, (24)	China	Diagnostic and Statistical Manual of Mental Disorders (DSM) + Chinese Classification and Diagnostic Criteria of Mental Disorders (CCMD)+ HAMD	2, RCT	30	27.98 ± 5.01	2.23 ± 0.43	EA + Mirtazapine	baihui(DU20) yintang(EX-HN3)	30min	7 times a week for 6 weeks	28	27.82 ± 5.2	2.18 ± 0.69	Mirtazapine	⑤⑪	1.5
Xu Feng, (25)	China	Diagnostic and Statistical Manual of Mental Disorders (DSM) + clinical manifestations	4, RCT	51	28.02 ± 6.1	NM	EA + Xiaoyaosan	taichong(BL15) qimen(LA14) neiguan(PC6) tanzhong(RN17) guanyuan(RN4) shenshu(BL23)	30min	6 times a week for 6 weeks	50/50/50	29.43 ± 4.18/30.43 ± 3.43/30.07 ± 3.34	NM	EA/Xiaoyaosan/placebo control	③④⑤	1.5
Xu SJ, (26)	China	Diagnostic and Statistical Manual of Mental Disorders (DSM) + clinical manifestations	2, RCT	35	20-45	NM	EA	baihui(DU20) yintang(EX-HN3) Genital area (a 2-centimeter straight line parallel to the anterior and posterior midline upward from the frontal horn)	30min	5 times a week for 6 weeks	35	20-45	NM	blank control	①②⑤⑪	1.5
Zhang WF, (27)	China	Diagnostic and Statistical Manual of Mental Disorders (DSM)	3, RCT	43	27 ± 1	0.85 ± 0.4	EA	auricular point	30-40 min	7 times a week for 4 weeks	39/43	27 ± 2/27 ± 2	0.8 ± 0.25/0.75 ± 0.28	Embedding needle therapy/Venlafaxine	⑤	1
Chung KF, (28)	China	Diagnostic and Statistical Manual of Mental Disorders (DSM) + HAMD	2, RCT	10	35.37 ± 4.7	NM	EA	baihui(DU20) yintang(EX-HN3) sishencong(EX-HN1) toulinqi(GB15) shuaigu(GB8) taiyang(EX-HN5) touwei(ST8) sanyinjiao(SP6) taichong(LR3) shenmen(HE7) neiguan(PC6)	30min	2 times a week for 4 weeks	10	34.47 ± 2.2	NM	sham EA	①②⑤⑪⑬	1
Xu YQ, (29)	China	EPDS + clinical manifestations	2, RCT	29	32.9 ± 4.25	8 ± 5.93	EA	yintang(EX-HN3) baihui (DU20) xinshu(BL15) ganshu(BL18) geshu(BL17) shenshu(BL23)	30min	2 times a week for 6 weeks	18	33 ± 4.08	5.37	sham EA	①②⑤⑪⑭⑮⑯	1.5
Wei HY, (30)	China	clinical guidelines and diagnostic essentials + clinical manifestations	2, RCT	45	28. 51± 3. 64	18. 16 ± 2. 68	EA	yintang(EX-HN3) baihui (DU20) sishencong(EX-HN1) zusanli(ST36) taichong(LR3) neiguan(PC6) sanyinjiao (SP6)	30min	3 times a week for 8 weeks	45	28. 92 ± 3. 51	18. 84 ± 2. 31	sham EA	③④⑤⑪⑰⑱⑲⑫	2
Xu Fang, (31)	China	Diagnostic and Statistical Manual of Mental Disorders (DSM) + clinical manifestations	4, RCT	50	29.43 ± 4.18	NM	EA	taichong(LR3) qimen(LA14) neiguan(PC6) tanzhong(RN17) guanyuan(RN4) shenshu(BL23)	30min	7 times a week for 6 weeks	51/50/50	28.02 ± 6.11/30.43 ± 3.43/30.07 ± 3.34	NM	EA+ Yinaojieyufang/Yinaojieyufang/placebo control	③④⑤⑪	1.5

① HAMD, Hamilton Depression Rating Scale; ② EPDS, Edinburgh postnatal depression scale; ③ E2, estradiol; ④ P, progesterone; ⑤ total effective rate; ⑥ WHOQOL-BREF, World Health Organization Summary of quality of life measurements; ⑦ AA, arachidonic acid; ⑧ DHA, docosahexaenoic acid; ⑨ EPA, eicosapentaenoic acid; ⑩ VD, vitamin D; ⑪ adverse event; ⑫ NE, Norepinephrine; ⑬ HDRS17, 17-item Hamilton Depression Rating Scale; ⑭ SAS, Self-rating Anxiety Scale; ⑮SDS, Self-rating Depression sale; ⑯ BIMF, Barkin Index of Maternal Functioning; ⑰ PRL, Prolactin; ⑱ DA, dopamine; ⑲ 5-HT, 5-hydroxytryptamine.

### 2.4 Quality assessment

The quality of the retrieved studies was assessed independently by the two investigators, using revised Cochrane risk-of-bias tool for randomized trials (RoB 2) (32). The RoB 2 tool encompasses six aspects for assessing the risk of bias, which are randomization process, deviations from intended interventions, mising outcome data, measurement of the outcome, selection of the reported result, overall Bias. Each criterion was analyzed and categorized based on the level of risk of bias as low, some concerns, or high.

### 2.5 Statistical analysis

Data were analyzed using Review Manager (version 5.3) and Stata (version 17.0). Continuous data were processed using the inverse variance method to determine the mean difference (MD) and 95% confidence interval (CI). Using the Mantel–Haenszel method, the total response rate and AEs were incorporated to calculate the risk ratio (RR) and a 95% CI (33). The *I^2^* statistic was employed to assess the heterogeneity among the studies. The studies were classified based on the level of heterogeneity, with low, moderate, or high levels corresponding to *I^2^* values of 25–50%, 50–75%, or >75%, respectively (34). The fixed-effects model was used for meta-analysis if there was no statistical heterogeneity among the findings, while the random-effects model was used when there was statistical heterogeneity. A p-value of less than 0.05 indicated statistical significance. Significant heterogeneity was addressed through sensitivity analysis, subgroup analysis, or solely description.

## 3 Results

### 3.1 Overview of literature search

**Figure 1:**
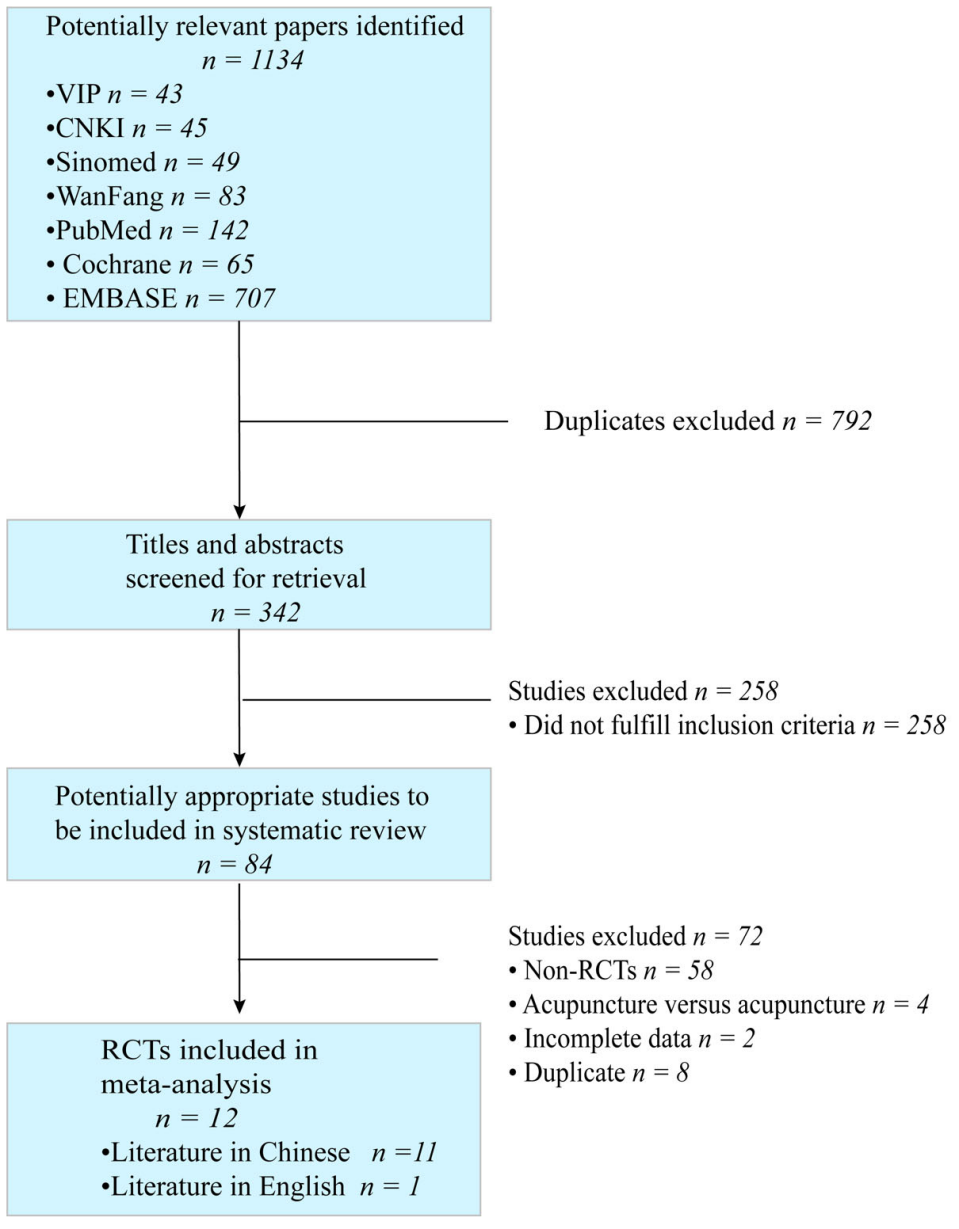
The process of review and selection of articles.

Initially, 1134 studies were retrieved for evaluation and screened according to their title and abstract content. Ultimately, 84 studies were chosen for further analysis. This study included 12 RCTs that investigated the use of EA for the treatment of PPD. Of these, 11 articles were in Chinese and 1 was in English. **Figure 1** illustrates the search and selection process.

### 3.2 Study characteristics

#### 3.2.1 Patients

A total of 571 patients from 12 studies (three of which were divided into two groups) were administered EA, while 793 patients in the control group received sham EA, TCM, antidepressants, no treatment (blank control group), or psychotherapy. Chinese researchers completed the entire work. It is worth watching that there is a lack of standardized diagnostic criteria for PPD. Among the studies included in our analysis, the CCMD was referenced in one study (24), while DSM was cited in eight studies (21, 23–28, 31). EPDS was utilized in four studies (20, 22, 23, 29), and the HAMD was employed in two studies (24, 28). Clinical manifestations were collectively considered in seven studies (21, 22, 25, 26, 29–31), and clinical guidelines and diagnostic essentials, specifically the International Classification of Diseases, 10th edition, were utilized in one study (30). The experimental group comprised a sample size ranging from 10 to 165 patients, while the control group included 10 to 171 patients. Except for one study (27) that did not specify the age of the patients, the participants in the research ranged in age from 20 to 45. Shown in **Table 1**.

#### 3.2.2 EA interventions

In terms of specific interventions, seven studies (23, 26–31) involved the use of EA alone, two studies (20, 24) involved a combined use of EA and antidepressants, and three studies (21, 22, 25) involved a combined use of EA and TCM. With regard to the selection of acupoints, the names of points were provided in all studies except one (27). The top four acupoints in terms of frequency of use were Baihui (GV20), Yintang (EX-HN3), Taichong (LR3), and Neiguan (PC6). The duration of treatment ranged from four to eight weeks.

#### 3.2.3 Control interventions

In the control group, four studies (20, 22, 24, 27) involved treatment with antidepressants, including paroxetine, sertraline hydrochloride tablets, venlafaxine, and mirtazapine. Furthermore, three cases involved sham EA (28–30), three cases used TCM (23, 25, 31), two cases used placebo control (25, 31), one study used a blank control (26), and one study involved psychotherapy (21).

#### 3.2.4 Outcome measures

Six studies utilized the HAMD (20, 21, 23, 26, 28, 29) scores to assess the alterations in PPD. The EPDS scale was utilized in five studies (21, 23, 26, 28, 29) to evaluate the condition of patients with PPD. Five studies (21, 23, 25, 30, 31) documented alterations in the levels of estradiol and progesterone in the bloodstream of patients with PPD. In addition, the study incorporated outcome indicators such as the score variations of the World Health Organization Summary of Quality of Life measurements, the Self-rating Anxiety Scale, and the Self-rating Depression Scale among patients with PPD. Furthermore, it examined alterations in the levels of dopamine, prolactin, arachidonic acid, vitamin D, docosahexaenoic acid, serotonin, eicosapentaenoic acid, and norepinephrine in the blood of patients with PPD. A total of nine studies (22, 24–31) evaluated the efficacy of the treatment (three of which were divided into two groups) (25, 27, 31). AEs were documented in six studies (24, 26, 28–31).

### 3.3 Risk assessment results

The Cochrane Handbook for Systematic Reviews of Interventions was utilized to access the risk of bias (32). Randomization was demonstrated in the 12 studies included. Eight studies utilized random number tables (21, 23, 25, 26, 28–31), while two studies employed the order of visits (24, 27). The details of the randomization process were not reported in two studies (20, 22). Three studies (21, 26, 29) documented the use of sealed envelopes for concealing the distribution of materials. Two studies (24, 27) achieved concealment through sequential numbering. Other studies did not document the methods for concealing the allocation. Baseline differences between all study intervention groups suggest no problems with the randomization process.

**Figure 2:**
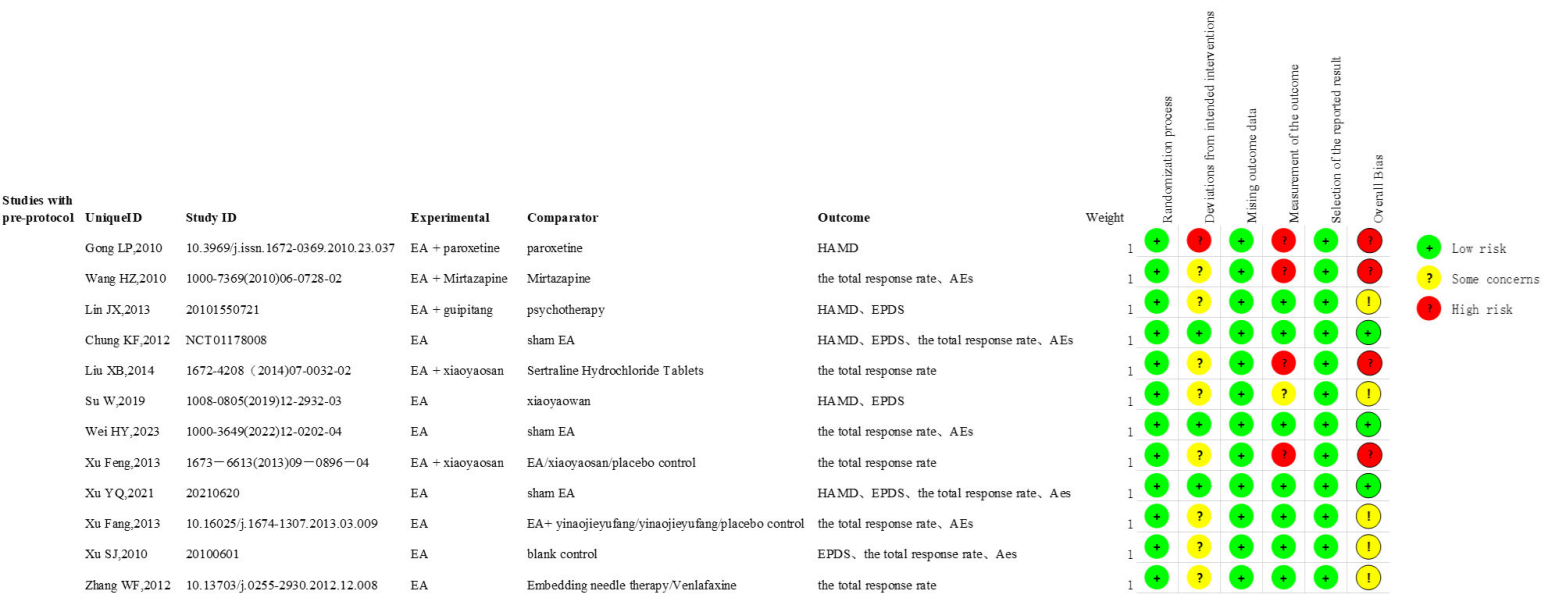
Risk of bias summary.

Blinding participants and practitioners is challenging in studies involving acupuncture interventions due to their specific study design characteristics. Nonetheless, three studies (28–30) attempted participant blinding to minimize performance bias. Five studies (21, 28–31) documented the use of blind evaluators, while the majority of other studies did not provide specific details on blinding procedures. All included RCTs maintained data integrity, and no selective reporting of results was identified. **Figure 2**, **Supplementary Figure S1** illustrates the quality assessment findings documented in this study.

### 3.4 Meta-analysis findings

#### 3.4.1 HAMD

**Figure 3:**
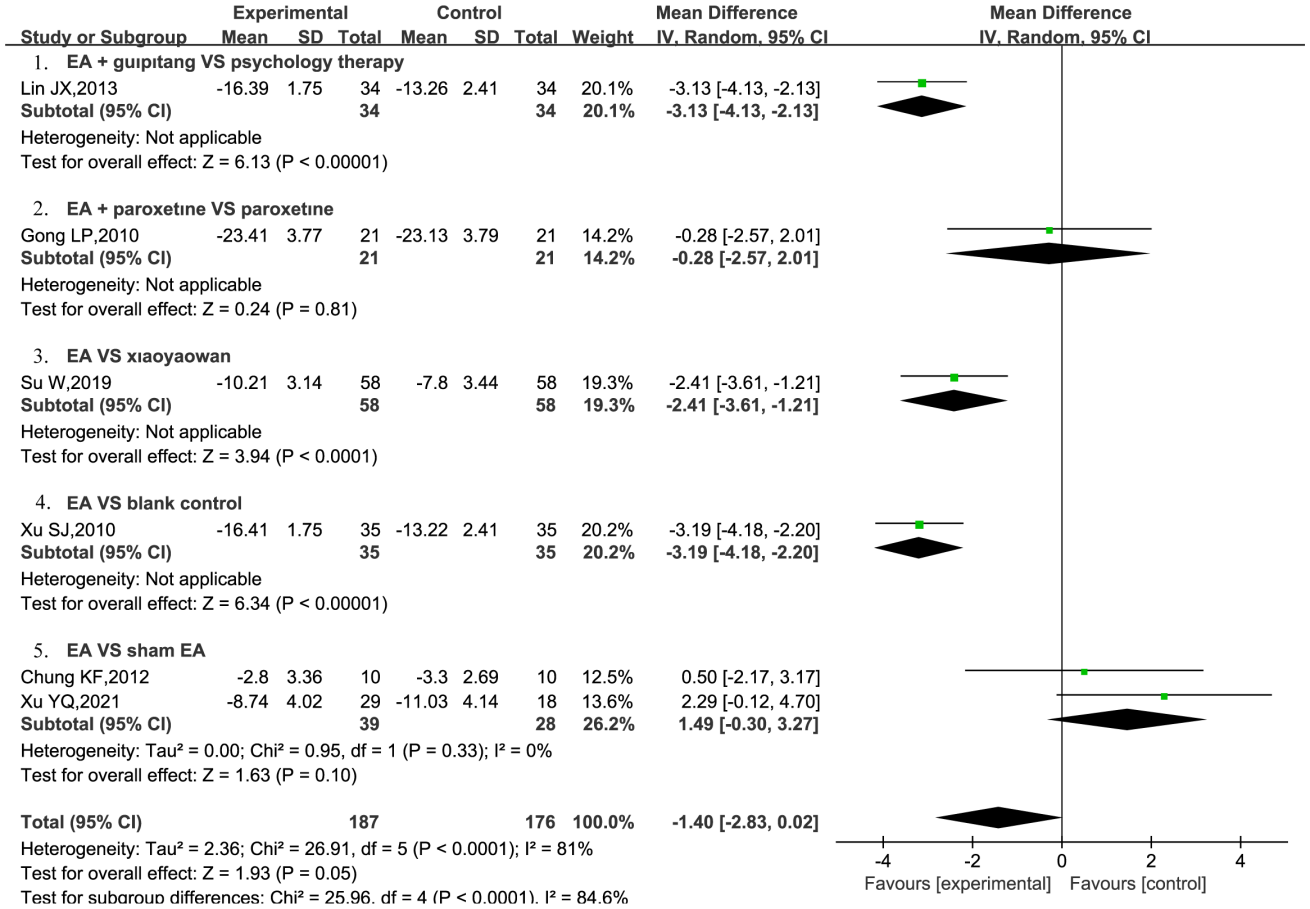
EA on HAMD scores in patients with PPD.

A total of six RCTs (20, 21, 23, 26, 28, 29) were included to assess the impact of EA on HAMD scores in patients with PPD. Furthermore, two RCTs (28, 29) provided a comparison between EA and sham EA. The results of the meta-analysis showed that EA was not more effective than sham EA in reducing HAMD scores, showing low heterogeneity (MD = 1.49, 95% CI = [−0.30, 3.27], *I^2^* = 0%). However, these results were not statistically significant (P = 0.1). However, a solitary study indicated that EA demonstrated greater efficacy compared to a blank control (MD = −3.19, 95%CI = [−4.18, −2.2], P < 0.00001) (26), and was also found to be more effective than Xiaoyaowan in reducing HAMD scores (MD = −2.41, 95%CI = [−3.61, −1.21], P < 0.0001) (23). Additionally, EA combined with Guipitang was found to be more effective than psychotherapy alone in reducing HAMD scores (MD = −3.13, 95% CI = [−4.13, −2.13], P<0.0001) (21) (**Figure 3**).

#### 3.4.2 EPDS

**Figure 4:**
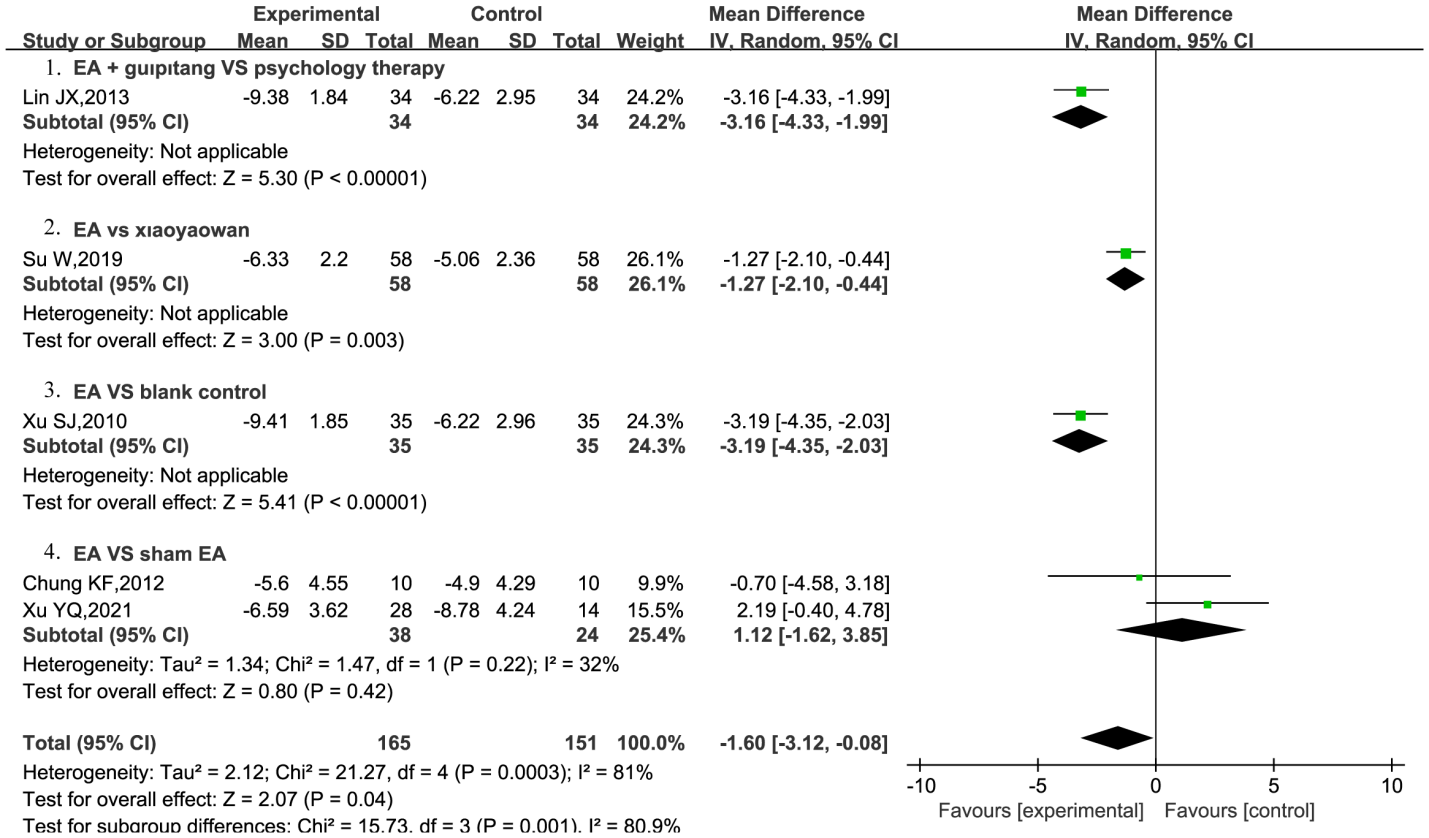
EA on EPDS scores in patients with PPD.

Five RCTs (21, 23, 26, 28, 29) were included to assess the effect of EA on EPDS scores in patients with PPD. Among these, two RCTs (28, 29) compared EA with sham EA. The results indicated that EA did not demonstrate superiority over sham EA in reducing EPDS scores, showing low heterogeneity (MD = 1.12, 95% CI = [−1.62, 3.85], *I^2^* = 32%), and the results were not statistically significant (P = 0.42). Only one study reported that EA outperformed the blank control in lowering EPDS scores (MD = −3.19, 95%CI = [−4.35, −2.03], P < 0.00001) (26). Moreover, EA was better than Xiaoyaowan in reducing EPDS scores (MD = −1.27, 95%CI = [−2.10, −0.44], P = 0.003) (23). Moreover, EA combined with Guipitang was found to be more effective than psychotherapy in reducing EPDS scores (MD = −3.16, 95% CI = [−4.33, −1.99], P<0.0001) (21) (**Figure 4**).

#### 3.4.3 Total response rate

A total of nine RCTs (22, 24–31) were included, and the total response rate was reported, with three RCTs (25, 27, 31) divided into two groups. The meta-analysis revealed total response rate of the EA intervention was significantly higher compared to other therapies, with statistically significant results (RR = 1.23, 95%CI = [1.08, 1.40], P = 0.002) and moderate heterogeneity (*I^2^* = 60%). Subsequent sensitivity analysis revealed that after excluding the study by Liu (22), the heterogeneity across the remaining studies decreased (*I^2^* = 22%), indicating a robust model.

**Figure 5:**
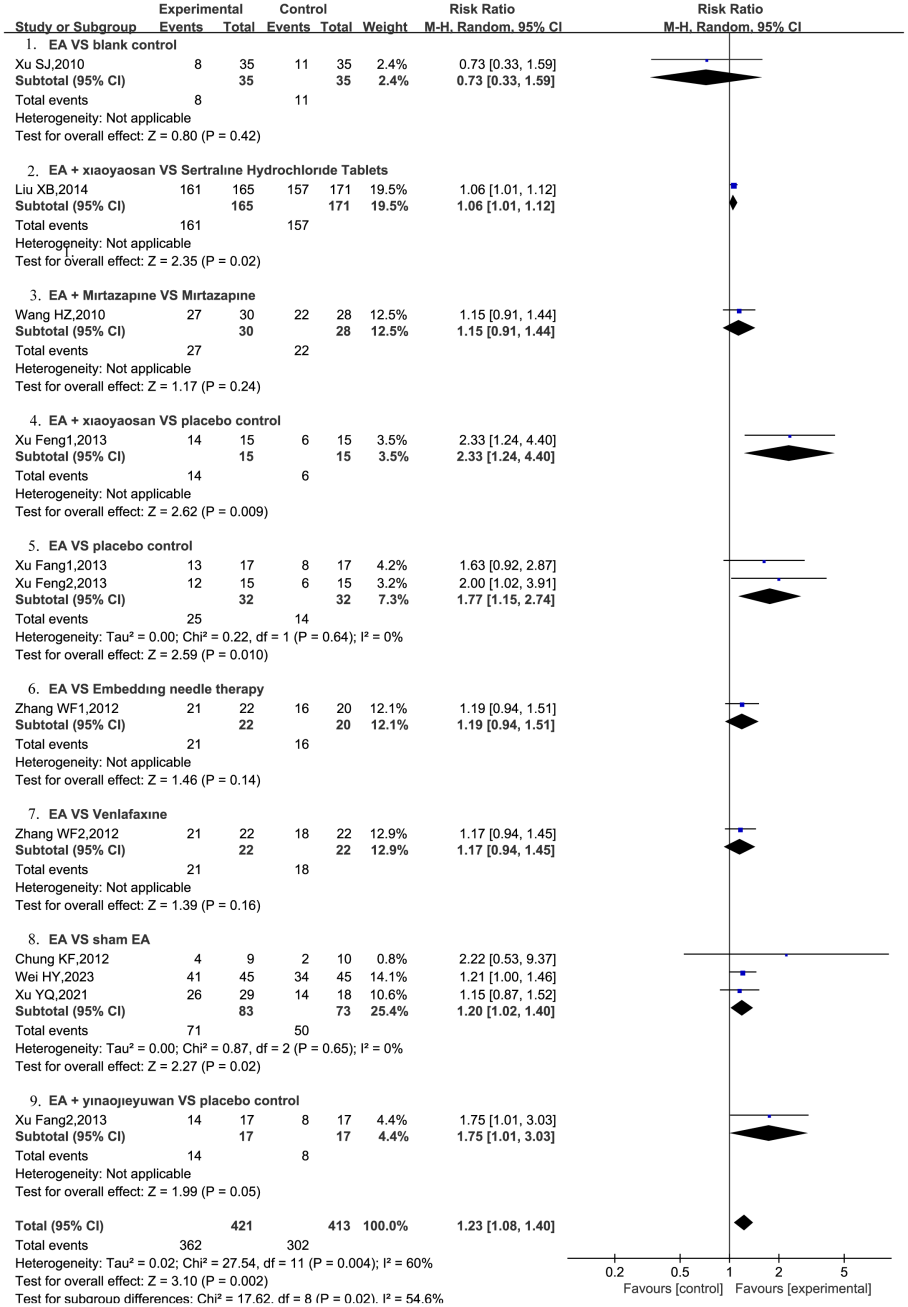
EA on total response rate in patients with PPD.

The comparison between EA and placebo control was reported in two RCTs (25, 31), whereas the comparison between EA and sham EA was reported in three RCTs (28–30). Furthermore, only one RCT each reported on EA + Xiaoyaosan vs. sertraline hydrochloride tablets (22), EA + mirtazapine vs. mirtazapine alone (24), EA + Xiaoyaosan vs. placebo control (25), EA vs. embedding needle therapy (27), EA vs. venlafaxine (27), EA + Yinaojieyufang vs. placebo control (31), and EA vs. blank control (26). The meta-analysis indicated that EA combined with Xiaoyaosan demonstrated superiority over sertraline hydrochloride tablets (RR = 1.06, 95%CI = [1.01, 1.12], P = 0.02) (22), Additionally, EA combined with Xiaoyaosan was found to be more effective than placebo control (RR = 2.33, 95% CI = [1.24, 4.40], P = 0.009) (25). EA + Yinaojieyufang was also found to be significantly more effective than placebo control (RR = 1.75, 95% CI = [1.01, 3.30], P = 0.05) (31). EA was found to be significantly more effective than placebo control (RR = 1.77, 95% CI = [1.15, 2.74], P = 0.01, *I^2^ =* 0%) (25, 31) as well as sham EA (RR = 1.2, 95% CI = [1.02, 1.40], P = 0.02, *I^2^* = 0%) (28–30), with the results indicating statistical significance and low heterogeneity, as shown in **Figure 5**.

#### 3.4.4 Adverse events

**Figure 6:**
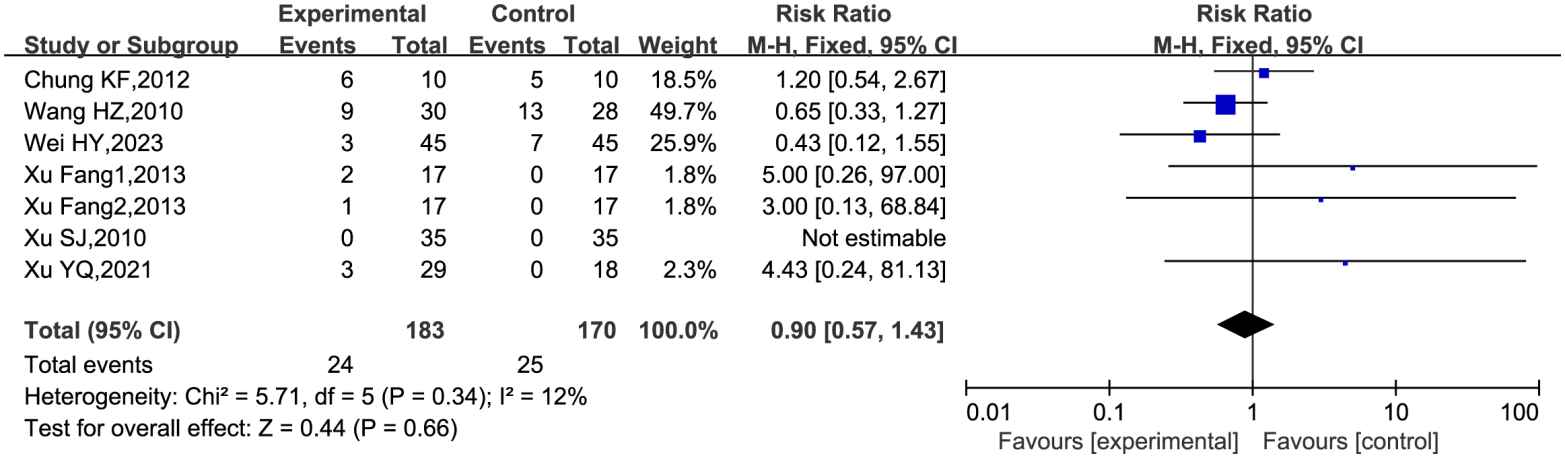
Forest map of AEs.

Six RCTs (24, 26, 28–31) included in the analysis reported AEs, with one RCT being divided into two groups (31). The meta-analysis indicated a low incidence of AEs associated with EA (RR = 0.9, 95%CI = [0.57, 1.43], P = 0.66, *I^2^* = 12%), with low heterogeneity among the studies. The primary adverse reactions associated with EA were pain and bruising at the site of needle insertion, as shown in **Figure 6**.

### 3.5 Detection of publication bias

**Figure 7:**
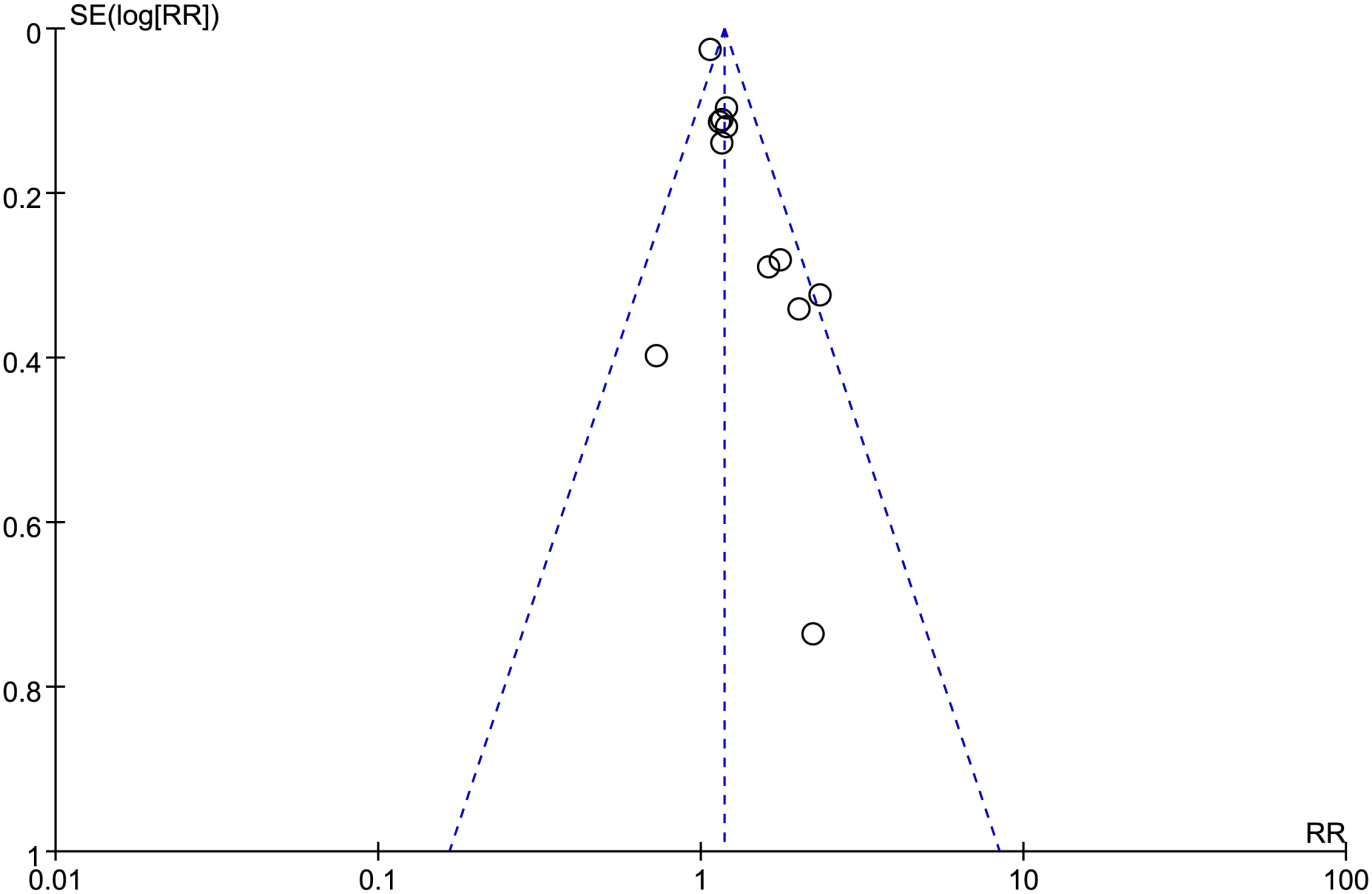
The funnel plot of the total response rate.

In this study, the researchers assessed publication bias by examining indicators that involved more than 10 studies. The Egger test, applied to the included literature assessing the total response rate of EA for PPD, suggested publication bias (P = 0.0011, < 0.05). Simultaneously, the funnel plot analysis also showed asymmetry, which also indicated the existence of publication bias in this study, as shown in **Figure 7**.

## 4 Discussion

Acupuncture plays a significant role in complementary and alternative medicine (35), and has been utilized for millennia to treat illnesses and restore the body’s equilibrium through the stimulation of acupoints (36). EA enhances the effect of acupuncture through electrical stimulation, has made significant advancements in treating depression. Research indicates that EA may achieve its therapeutic effects on depression by modulating brain network regulation (37). One study has demonstratedhat EA could potentially modulate depression by up-regulating theexpression of basic fibroblast growth factor, thereby influencing astrocyte proliferation (38). The mechanism of EA treatment for PPD may involve regulating hormone secretion in the hypothalamic-pituitary-gonadal axis and plasma estradiol levels (39). The mechanism of action may also involve improving the distribution of intestinal flora in PPD patients (40, 41).

Previous meta-analyses (42, 43) have shown the potential benefits of acupuncture, including manual acupuncture, moxibustion and other techniques for PPD (44–46), but did not specifically discuss the efficacy and safety of EA in PPD treatment. Therefore, this meta-analysis aims to further explore the efficacy and safety of EA in treating PPD.

The results of this study indicate that the EA group showed significantly higher overall response rates in comparison to both the placebo group and the sham EA group. These results suggest that EA may possess superior clinical efficacy to a certain degree. However, there was no discernible advantage of EA over sham EA in reducing HAMD and EPDS scores among patients with PPD. While the evidence remains inconclusive, EA has exhibited promise in the treatment of PPD. For instance, in comparison to the blank control group (26) and TCM Xiaoyaowan (23), EA showed better efficacy in reducing HAMD and EPDS scores. Another independent study (21) highlighted the advantages of integrating EA with TCM Guipitang, compared to psychotherapy, in decreasing HAMD and EPDS scores in PPD patients. Although the findings of our meta-analysis suggest that EA did not lead to a significant reduction in depression scores, it did show a higher overall response rate compared to both placebo and sham EA groups. Furthermore, our investigation demonstrated a minimal occurrence of AEs linked to EA; hence, EA remains a safe and effective treatment for postpartum depression.

Nevertheless, it is crucial to acknowledge a few limitations in this meta-analysis. Firstly, it is important to note that certain study results exhibited high heterogeneity and are subject to publication bias. Additionally, the competitive nature of academic research may result in the unavoidable exclusion of negative research results. Another limitation is that all studies were carried out in a single country, namely China. Furthermore, the limited number of included studies and their relatively small sample sizes further affected the accuracy of our meta-analysis. Ultimately, the majority of studies failed to provide details on allocation concealment and blinding techniques. This lack of rigorous methodological transparency can introduce bias into the study results and weaken the validity of the conclusions. Addressing these methodological shortcomings in future research is essential to improve the quality and reliability of RCTs in this field.

In summary, the existing evidence indicates that EA shows promise in effectively and safely treating PPD. The above conclusions should be validated through additional high-quality studies, given the limitations pertaining to the quantity and quality of the studies included in this meta-analysis.

## Data availability statement

The original contributions presented in the study are included in the article/**Supplementary Material**. Further inquiries can be directed to the corresponding author.

## Author contributions

XF: Writing – original draft, Writing – review & editing. XW: Writing – review & editing, Conceptualization. WZ: Data curation, Software, Writing – review & editing. JH: Funding acquisition, Writing – review & editing. XG: Writing – review & editing, Formal analysis, Visualization.
