# Supplementary material for: Efficacy and safety of electroacupuncture in patients with postpartum depression: a meta-analysis
Source: Front Psychiatry. 2024 Jul 11;15:1393531. doi: 10.3389/fpsyt.2024.1393531 (PMC11270539; doi:10.3389/fpsyt.2024.1393531)
Supplement: Supplementary file 3 [file Table_1.docx]

**Pubmed search strategy**

#1 electroacupuncture[MeSH]

#2 (electroacupunct* or electro acupunct*)[Title/Abstract]

#3(electrical acupuncture or Electric acupuncture or Galvanoacupuncture or EA)[Title/Abstract]

#4 #1 OR #2 OR #3

#5 Depression, Postpartum[MesH]

#6 (postpartum or post partum or postnatal or post natal or puerper*)[Title/Abstract]

#7 (depress* or dysthymi* or adjustment disorder* or mood disorder* or affective disorder* or affective symptom*)[Title/Abstract]

#8 #5 OR #6 OR #7

#9 #4 AND #8
